# Effects of Testosterone Replacement Therapy on Glycolipid Metabolism Among Hypogonadal Men withT2DM: A Meta-Analysis And System Review Of Randomized Controlled Trials

**DOI:** 10.1016/j.esxm.2021.100403

**Published:** 2021-07-20

**Authors:** Xiaowei Yu, Zhentong Wei, Yanhong Liu, XiaoYuan Zhang, Qun Wang

**Affiliations:** 1 Department of Reproductive Medicine, Department of Prenatal Diagnosis, The First Hospital of Jilin University, Changchun, Jilin, China; 2 Department of Obstetrics and Gynecology, The First Hospital of Jilin University, Changchun, Jilin, China

**Keywords:** Hypogonadal, TRT, T2DM, Metabolism

## Abstract

**Introduction:**

Testosterone can improve glucose metabolism through multiple cellular mechanisms. However, it remains unclear as to whether hypogonadal men with type 2 diabetes mellitus (T2DM) can benefit from testosterone replacement therapy (TRT).

**Aims:**

To assess the relative effect of TRT on glycolipid metabolism among hypogonadal men with T2DM.

**Methods:**

: Electronic literature searches of the Cochrane Library, PubMed, MEDLINE, and EMBASE databases were conducted, up to the end of October 2020. Only studies that used randomized controlled trials (RCTs) were included in our systematic review. Main outcome measures From these studies, we extracted certain outcomes including changes in insulin resistance, glucose metabolism, and lipid parameters.

**Results:**

There were a total of 8 studies that met our criteria. Four of these studies either did not have a consistent treatment strategy, or the control groups used untreated patients rather than patients that had been given a placebo. Thus, results from these four studies contributed to the variability in treatment outcomes. In four of the examined RCTs, there was no change in either the dose or the type of antidiabetic medication prescribed. Based on the homeostatic model assessment of insulin resistance, the pooled WMD was −0.34, 95% confidence interval (CI; −1.02, 0.34), *P* = .33; For fasting plasma glucose, the pooled WMD was −0.27, 95% CI (−1.02, 0.48), *P* = .48, the pooled WMD for HbA1c% was −0.00, 95% CI (−1.08, 1.08), *P* = 1.00.

**Conclusions:**

Although certain RCTs showed that TRT improved insulin resistance and glycolipid metabolism when compared with the placebo or untreated control groups, these findings may partly be due to changes in antidiabetic therapy during the course of the study. In the current meta-analysis, analyses showed that TRT did not significantly improve insulin resistance or glycolipid metabolism. Future studies need to be rigorous in design and delivery, and comprehensive descriptions of all aspects of their methods should be included to further enable a more accurate appraisal and interpretation of the results.

**Yu X, Wei Z, Liu Y, et al. Effects of Testosterone Replacement Therapy on Glycolipid Metabolism Among Hypogonadal Men with T2DM: A Meta-Analysis And System Review Of Randomized Controlled Trials. Sex Med 2021;9:100403.**

## INTRODUCTION

Hypogonadism is a clinical syndrome caused by the disruption of the hypothalamic-pituitary-testicular axis, such that the testes fail to produce physiological levels of testosterone and defects in spermatogenesis occurs.^1^ Diagnosis of male hypogonadism requires both a persistent androgen deficiency and the presence of clinical symptoms (such as declines in sexual function, osteoporosis, muscle strength, and metabolic disorders).^2^ Nearly 30% of type 2 diabetes mellitus (T2DM) patients were found to have lower levels of circulating testosterone.^3^^,^^4^ In addition, androgen suppression treatment was found to be associated with an increased incidence of developing T2DM in younger men with hypogonadism.^5^, ^6^, ^7^, ^8^

Testosterone has been shown to be a key regulator in the maintenance of metabolic homeostasis, and some researchers thus hypothesized that TRT may improve glucose metabolism. For instance, in a large cohort registry study spanning eight years,^9^ 316 men with pre-T2DM and testosterone deficiency were treated with 1,000 mg of testosterone every 12 weeks. The results showed that the men who underwent TRT had significant improvements in their anthropometric measurements, glycemic control, and lipid levels when compared with the control group. All TRT patients had a glycated hemoglobin A1c (HbA1c) of < 6.5, and 90%of them also achieved normal glucose regulation with a HbA1c of < 5.7, compared with only 1% of the untreated group. However, this study did not mention whether antidiabetic drugs were administered to the patients over the 8-year period, which weakens the strength of the obtained evidence. Numerous randomized controlled trials (RCTs) have been conducted to evaluate the effects of TRT on hypogonadal men with T2DM, but unfortunately, the limited evidence available remains controversial.^10^, ^11^, ^12^, ^13^, ^14^, ^15^, ^16^, ^17^, ^18^, ^19^, ^20^ While various studies have demonstrated that TRT improves insulin resistance and glycolipid metabolism in individuals withT2DM,^10^^,^^11^^,^^14^^,^^16^^,^^18^^,^^19^ other RCTs did not show such an association.^12^^,^^13^^,^^15^^,^^17^^,^^20^

Previous meta-analyses of RCTs showed that TRT seemed to improve glycemic control as well as the fat mass of hypogonadal men with T2DM.^21^, ^22^, ^23^, ^24^ However, these meta-analyses are characterized by several limitations. For instance, some RCTs were conducted without a placebo control group, while other studies included metabolic syndrome patients as participants. Moreover, all the meta-analyses included studies in which antidiabetics and lipid-lowering therapies, were administered to participants in addition to the TRT. Hence, it is difficult to assess whether the improvements observed were due to TRT or the other simultaneous treatments. The presence of these limitations thus restricts the generalizability of the conclusions drawn. Furthermore, since the publication of these meta-analyses, additional studies in this field have been published,^14^^,^^19^^,^^20^ which offers additional important information for the accurate appraisal of the relationship between testosterone levels and both insulin resistance and glycolipid metabolism.

However, there have been no consensus on TRT for hypogonadism in T2DM men. Thus, we integrated all available qualified RCTs and conducted a meta-analysis of studies the treatment strategy needs to be maintained throughout the entire study, to assess the metabolic effects of TRT on hypogonadal men with T2DM.

## Materials and Methods

We adhered to the Preferred Reporting Items for Systematic Reviews and Meta-analysis (PRISMA) statement. The present study was also registered at PROSPERO (registry number: CRD42021243806). The study was exempted from Institutional Review Board approval given that it did not involve any human intervention.

### Literature Search

To identify all relevant published studies, a literature search of Pubmed, Cochrane Library, and Google Scholar was performed from their inception up until October 2020. Trial registries (http://clinicaltrials.gov and www.who.int/trialsearch) were also searched. The parameters of the primary search combined terms and descriptors related to “Type 2 Diabetes”, “testosterone OR testosterone replacement”, and “hypogonadism OR androgen deficiency syndrome”, with the filters “human” and “clinical trial” in any language. Furthermore, for studies to be selected, they had to report on glycolipid metabolism and whether or not antidiabetic medications of any type were used. For the advanced search, the parameter of the article type was set to search strictly for only RCTs. Manual searches of the reference lists of the articles included in the present study were also performed to identify additional relevant studies.

### Eligibility Criteria and Study Selection

Articles were included if full texts were available, human participants were enrolled, and they were not review articles. Studies that included the following were selected: 1) Studies were RCTs that assessed the efficacy of the combined treatment of antidiabetic medication and TRT or TRT monotherapy on glycolipid metabolism in hypogonadal men with T2DM; 2) An assessment of glycolipid metabolism as one of the study outcomes; 3) Definitive diagnosis of hypogonadism (both a persistent androgen deficiency and the presence of clinical symptoms) and T2DM (fasting plasma glucose over 7.0 at baseline and/or over 11.1 after a 2-h, 75-g oral glucose tolerance test and an elevated level of HbA1c) Exclusion criteria encompassed studies that had: 1) Unclear diagnoses of hypogonadism (not Testosterone Deficiency) and T2DM (not Metabolic Syndrome); 2) T2DM patients with normal testosterone levels; 3) No placebo or other types of control groups; 4) Insufficient data for pool estimation. Two authors (QW and ZTW) independently assessed all of the abstracts retrieved from the search and obtained full manuscripts of the papers that met the selection criteria. These two authors also evaluated the studies’ eligibility and subsequently extracted the required data. Any discrepancies in study selection were solved through discussions and consensus between the two authors was required. If consensus could not be reached, a third author (XWY) was involved in the discussion and a decision was made based on consensus between two of the three authors (YHL and ZTW).

### Data Extraction

Two authors (QW and XYZ) independently extracted the data from the selected studies. The following characteristics were assessed for each study: i) Author and year of publication; ii) Definition of hypogonadism; iii) Type and duration of TRT; iv) Use of antidiabetic medications or lipid-lowering therapies; v) Consistency of antidiabetic treatment over the entire study period; vi) Setup of the control group. The risk of bias in several aspects including the study selection, performance, attrition, detection, and reporting bias, was assessed by using the Cochrane Collaboration's tool. In addition, other biases assessed in the present study primarily concerns participants’ demographics, including age, baseline testosterone levels, and type of antidiabetic treatment. The primary outcomes that were evaluated were glucose metabolism and insulin resistance. Glucose metabolism was assessed using fasting plasma glucose (FSG), fasting serum insulin (FSI), and HbA1c, while insulin resistance was calculated using the homeostatic model assessment of insulin resistance (HOMA-IR). Lipid metabolism was used as a secondary outcome, and was determined by assessing the levels of total cholesterol (TC), high-density lipoprotein (HDL), and low-density lipoprotein (LDL).

### Sensitivity Analysis and Risk of Bias

Sensitivity analyses using the leave-one-out approach indicated that the direction and magnitude of the combined estimates did not change markedly with the exclusion of individual studies, and signifies that the meta-analysis had good reliability. Due to the small number of included trials, funnel plot asymmetry tests to evaluate publication bias were not performed.

### Statistical Analyses

Statistical analyses were conducted using Review Manager (version 5.3; The Cochrane Collaboration, Oxford, UK). Associations between continuous variables were assessed using the weighted mean difference (WMD) and their 95% confidence intervals (CIs). Sensitivity analyses were conducted by excluding trials with outlying data points. Heterogeneity between trials was quantified using either the Q-test or I^2^. A random effect model was utilized due to the limited number of studies.^25^

## RESULTS

### Search Results and Study Characteristics

**Figure 1:**
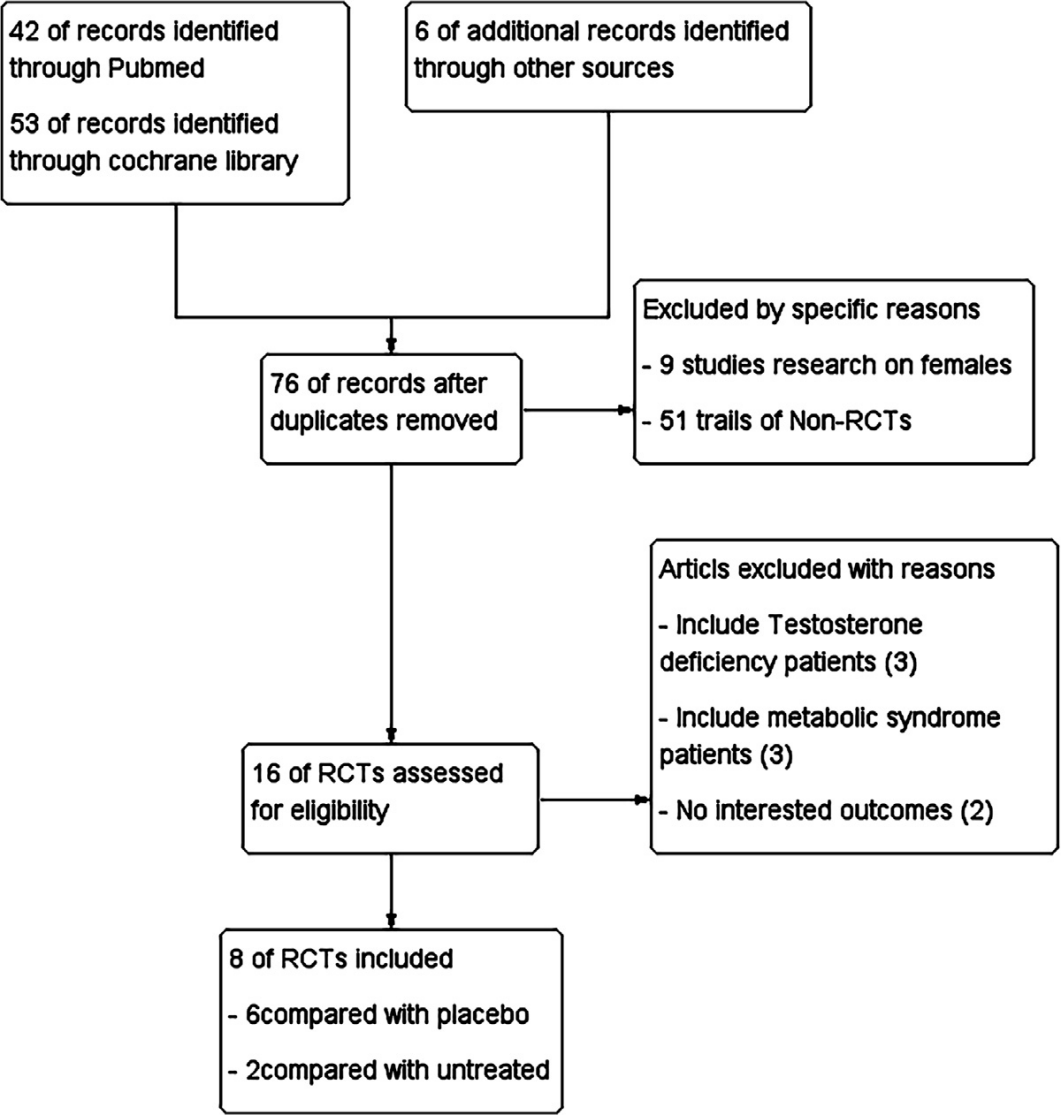
Trial identification and selection process. 257 × 270mm (300 × 300 DPI).

A total of 101 articles were retrieved based on the search parameters described above, and were first screened by their title and abstract. After excluding publications which failed to meet the inclusion criteria, the remaining 8RCTs were explored by the authors. The reasons for exclusion are listed in the flow chart in Figure 1. Eight RCTs (Table 1) were included in this systematic review in which hypogonadal men with T2DM were either subjected to TRT[13-15, 17, 18, 20] or untreated.^10^^,^^19^ In seven of the included studies,^10^^,^^13^, ^14^, ^15^^,^^17^^,^^18^^,^^20^ concomitant administration of antidiabetic medication was permitted and sustained throughout the study.

**Table 1:** Characteristics of the clinical studies included in the present meta-analysis

Author (year)	Hypogonadism diagnosis criteria	Patients included	T and dose	Antidiabetic drug %	lipid-lowering therapies%	Treatment consistency	control	Significant improvement Outcome	Trial duration weeks
Hackett 2014	with symptoms of hypogonadism	TT: 8.1 -12.0nmol/L	i.m. TU 1000mg at week 0, 6, 18	Insulin (23.7) Oral Antidiabetic (78.9) Diet (14.7)	76.4%	YES	Placebo	TC	21
Gopal 2010	with symptoms of hypogonadism	FT <64.8pg/ml	i.m. TC 200 mg per 2 weeks	Insulin (22.7) Oral Antidiabetic (68.2)	72.7%	YES Only patients hypoglycemia reduced insulin doses	Placebo	None	16
Kapoor 2006	with symptoms of hypogonadism	TT≤12.0nmol/L	i.m. Sutanon 200 mg per 2 weeks	Insulin (41.7) Oral Antidiabetic (58.3)	NA	YES Only patients hypoglycemia reduced insulin doses	Placebo	FSG, HbA1c, HOMA-I, TC	12
Boyanov2003	have symptoms of andropause or erectile dysfunction	TT≤15.1nmol/L	O‑TU 120 mg daily	Insulin (25) Oral Antidiabetic (75)	No	NA	untreated	FSG, HbA1c	12
Magnussen2016	With symptoms and signs suggestive of hypogonadism	TT≤7.3nmol/L	T‑gel 1% 50 mg daily	Oral Antidiabetic (Metformin only)	NA	YES	Placebo	HDL	24
Groti 2018	With three sexual symptoms: decreased sexual interest, absent or rare morning erections and erectile dysfunction	TT≤11.0nmol/L or FT <220pmol/l	i.m. TU 1000mg at week 0, 6, Each 10 weeks after	Oral Antidiabetic (unspecified)	52.7%	NA	Placebo	FSI, HbA1c, HOMA-I	48
Jones 2011	with at least two symptoms of hypogonadism	TT≤11.0nmol/L or FT <255pmol/l	T‑gel 2% 60 mg per daily	Oral Antidiabetic (unspecified)	NA	YES First 6 months no therapy changes	Placebo	None	24
Khripun 2018	With at least two symptoms or complaints of sexual or psychological nature	TT<12.1nmol/L	T‑gel 1% 50 mg daily	Diet only	No	YES	untreated	FSG, FSI, HbA1c, HOMA-I, HDL	36

FSG = serum plasma glucose; FSI = fasting serum insulin; HDL = high‑density lipoprotein; HOMA-IR = Homeostatic model assessment of insulin resistance; i.m. = deep intramuscular injection; LDL = low‑density lipoprotein; NA = not available; O‑TU = oral testosterone undecanoate; T = testosterone; TC = Total cholesterol; TC = testosterone cyponate; NA = not available.

Specifically, three studies administered oral hypoglycemic medication, including Metformin monotherapy^20^ and other unclassified medications,^14^^,^^17^ but not insulin treatment. Five studies[13, 15, 17, 18, 20]explicitly stated that the consistency of the whole treatment strategy was maintained without any adjustment to the type and dose of the administered antidiabetic therapy. Out of these five studies, one presented data using the median (inter quartile range) values,^20^ therefore, it was excluded from the qualitative pool analysis.^13^^,^^15^^,^^17^^,^^18^ Antidiabetic therapy was restricted to dietary changes only in the remaining article.^19^ Treatment duration ranged from 3 to 12 months. Table 2 describes the main outcomes of the eight included articles.

**Table 2:** Primary variables used in the randomized controlled trials included in the present meta-analysis

Author (year)	Patiens (T/C)	Mean ofBMI/ Age	FSG (T/C)	FSI (T/C)	HbA1c (%)(T/C)	HOMA-IR (T/C)	TC (T/C)	HDL (T/C)	LDL (T/C)
Hackett 2014^A^	91/95	32 61	9.45±3.29 9.47±3.96	no significan	7.68±1.26 7.54±1.21	4.16±2.58 3.94±2.22	3.91±0.79 4.08±1.07∗	1.00±0.27 1.06±0.30	2.11±0.69 2.14±0.86
Gopal 2010^A^	11/11	24 44	155.25 ± 48.40 196.80 ± 69.02	14.54±9.86 16.02±19.31	6.25 ± 2.05 6.27 ± 2.67	5.88 ± 5.26 8.16 ± 10.49	171.17 ± 35.71 169.10 ± 60.48	37.34 ± 6.73 33.56 ± 13.16	124.50± 25.58 112.61± 52.81
Kapoor 2006^B^	12/12	33 64	7.38± 0.37 8.73± 0.61∗	11.76±1.76 12.36±2.13	−0.37±0.17∗	−1.73±0.67∗	4.83± 0.2 5.07± 0.17∗	0.97± 0.04 1.02± 0.04	2.74± 0.18 2.81± 0.17
Boyanov 2003^A^	24/24	31 57.5±4.8	6.0 ± 1.3 8.0 ± 2.4∗	NA	8.6 ± 1.0 9.9 ± 1.4∗	NA	5.42 ± 1.47 5.55 ± 1.46	1.21 ± 0.22 1.18 ± 0.23	3.60 ± 1.25 3.69 ± 1.30
Magnussen2016^D^	20/19	30 61	7.3 (6.7-8.0) 7.2 (6.6-8.0)	81.2 (63.5-103.7) 102.5 (79.7-131.9)	6.7 (6.4-7.0) 6.6 (6.2-6.9)	3.8 (2.9-5.0) 4.8 (3.6-6.3)	3.7 ± 1.0 3.9 ± 0.9	0.95 (0.84-1.0) 1.00 (0.93-1.0)∗	2.1 (1.8-2.4) 2.0 (1.7-2.4)
Groti 2018^A^	28/27	33 60	8.83 ± 1.21 9.47 ± 1.31	17.51 ± 10.7 24.38 ± 12.82∗	7.18 ± 0.81 7.65 ± 0.70∗	6.81 ± 4.18 10.18 ± 5.60∗	4.62 ± 0.63 4.89 ± 0.74	1.04 ± 0.21 1.07 ± 0.29	2.70 ± 0.59 2.54 ± 0.54
Jones 2011^A^	68/69	32 60	8.91± 3.31 9.09±2.97	121.61± 102.01 136.40±117.44	no significan	no significan	4.44 ± 1.12 4.57 ± 0.97	1.09 ± 0.27 1.21 ± 0.26	2.60 ± 0.85 2.55 ± 0.70
Khripun 2018^C^	38/38	34 53	6.3 (2. 2) 8.8 (5.0)∗	17.5 (7.6) 26.2 (13.9)∗	6.7 (1.9) 8.4 (3.1)∗	6.3 (4.8) 11.6 (5.9)∗	5.1 (1.2) 5.5 (1.8)	1.52 (0.13) 1.43 (0.13)∗	2.86 (1.04) 3.34 (1.32)

A mean± SD.

B mean± SE.

C median (IQR).

D mean (95% CI). BMI: body mass index; FSG: serum plasma glucose; FSI: fasting serum insulin; HOMA-IR: Homeostatic model assessment of insulin resistance; Totla cholesterol: TC; HDL: high density lipoprotein; LDL: low density lipoprotein; NA: not available; HbA1c: glycated hemoglobin A1c; T⁄C: testosterone ⁄ control; TT: total testosterone.

∗ : *P*<.05.

### Glycolipid Metabolismin TRT with Concomitant Administered Antidiabetics

Four of the included studies compared TRT with the concomitant administration of antidiabetic medication, such as oral antidiabetics and insulin (Table 1).^10^^,^^13^^,^^15^^,^^18^ The percentage of patients using insulin in these studies ranged from 22.7% to 49.6%. A placebo group was used as the control in three of the studies,^13^^,^^15^^,^^18^ while untreated patients were used as controls in the remaining studies.^10^ In terms of the consistency of the treatment strategies, one study made no mention of this factor and reported that TRT conferred beneficial effects on insulin resistance and glucose metabolism.^10^ The remaining three studies explicitly stated that the dose and type of antidiabetic medication was maintained throughout the study, and one of these three studies demonstrated that TRT was associated with improvements in insulin resistance (HOMA-IR), glucose metabolism (FSG, HbA1c), and TC[18]. The other two studies found that TRT was not associated with improvements in insulin resistance or glucose metabolism.^13^^,^^15^ (Tables 1 & 2)

### *Glycolipid Metabolism of TRT with Oral*Antidiabetics

Three studies investigating the effect of TRT on glycolipid metabolism in hypogonadal men with T2DM used a placebo group as the control, where men were generally treated with either Metformin^20^ or unspecified oral antidiabetics.^14^^,^^17^ Two studies^17^^,^^20^ which explicitly stated that there were no changes in antidiabetic treatment either during the entire study^20^ or during the first six months,^17^ found that apart from improvements in HDL, there were no other improvements in insulin resistance or glycolipid metabolism with TRT. In the remaining study which made no mention of treatment consistency in terms of antidiabetic medications, TRT was associated with improvements in HOMA-IR and glucose metabolism (FSI, HbA1c), but not in lipid metabolism (TC, HDL, LDL).^14^

### Meta-analyses of the “High-quality” RCTs

**Figure 2:**
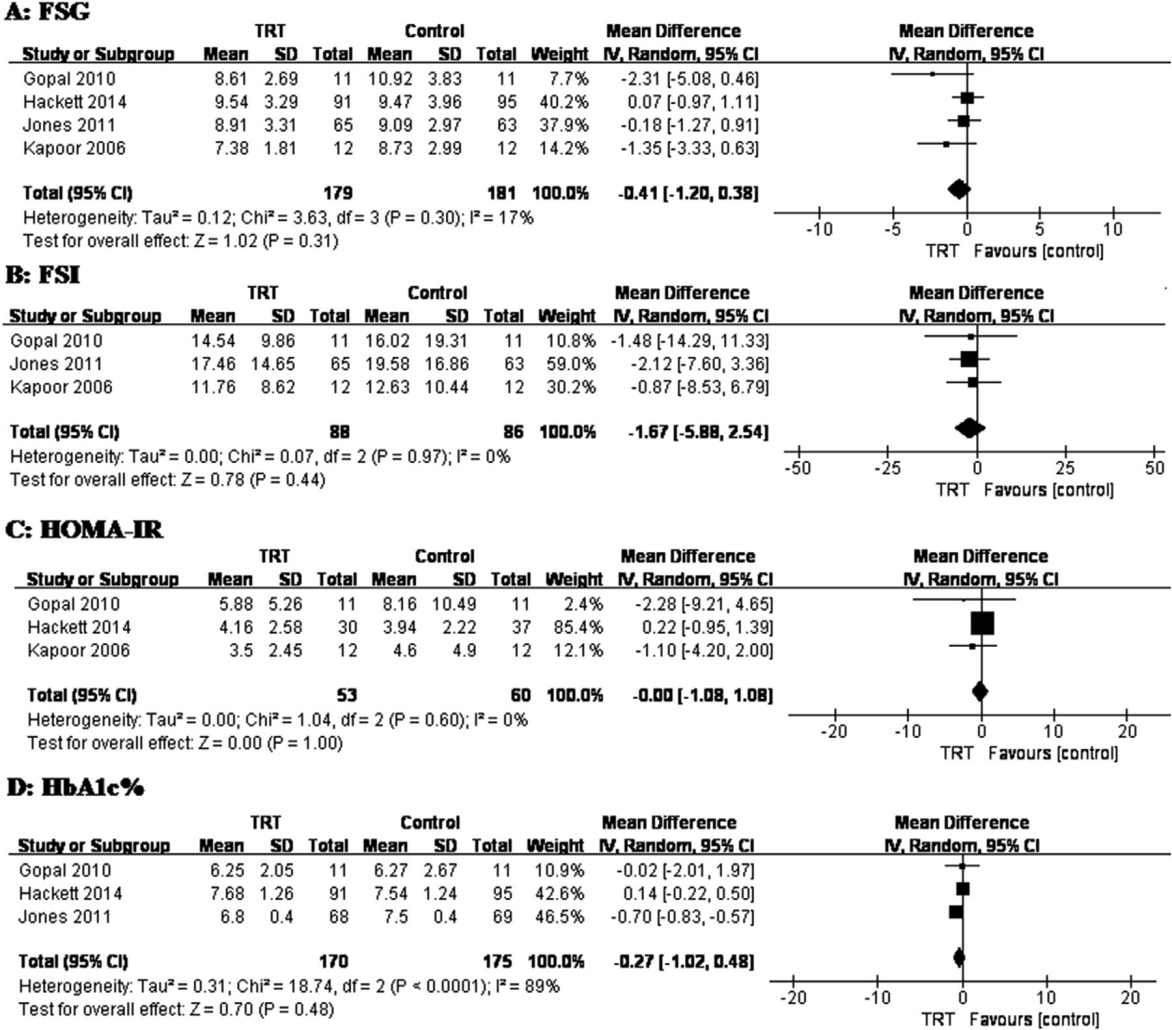
Weighted differences (with 95%CI) of mean fasting serum glucose (A:FSG), fasting serum insulin (B:FSI), HOMA-IR (C) and HbA1c (%) (D) between testosterone and control groups. 299 × 270mm (300 × 300DPI).

There were four “High-quality” RCTs in the present study,^13^^,^^15^^,^^17^^,^^18^ which are defined as such by the following two conditions. First, the treatment strategy needs to be maintained throughout the entire study. Second, the control group must consist of patients administered with placebos, instead of untreated patients. We performed two meta-analyses as part of this review. For glucose metabolism, a pooled analysis of FSG, FSI, HbA1c, as well as insulin resistance using HOMA-IR was conducted. The pooled analysis of FSG included 360 patients, of which 179 underwent TRT and 181 acted as controls, resulting in a pooled WMD of −0.41,95% CI (−1.02, 0.38), *P* = .31 (Figure2A). The FSI analysis was conducted with 174 patients, consisting of 88 who underwent TRT and 86 who acted as controls, resulting in a pooled WMD of −1.67,95% CI (−5.88, 2.54), *P* = .44 (Figure2B). The HOMA-IR was estimated using either the original HOMA1 equation^13^^,^^18^ or the computer-based HOMA2 model.^15^^,^^17^ HOMA-IR analysis included 345 patients, consisting of 53 in the TRT group and 60 in the control group, resulting in a pooled WMD of 0.00, 95% CI (−1.08, 1.08), *P* = 1.00 (Figure2C). For the HbA1c% analysis, which consisted of 170 patients who underwent TRT and 175 patients who acted as controls, the pooled WMD was −0.41,95% CI (−1.02, 0.48), *P* = .48 (Figure2D).

**Figure 3:**
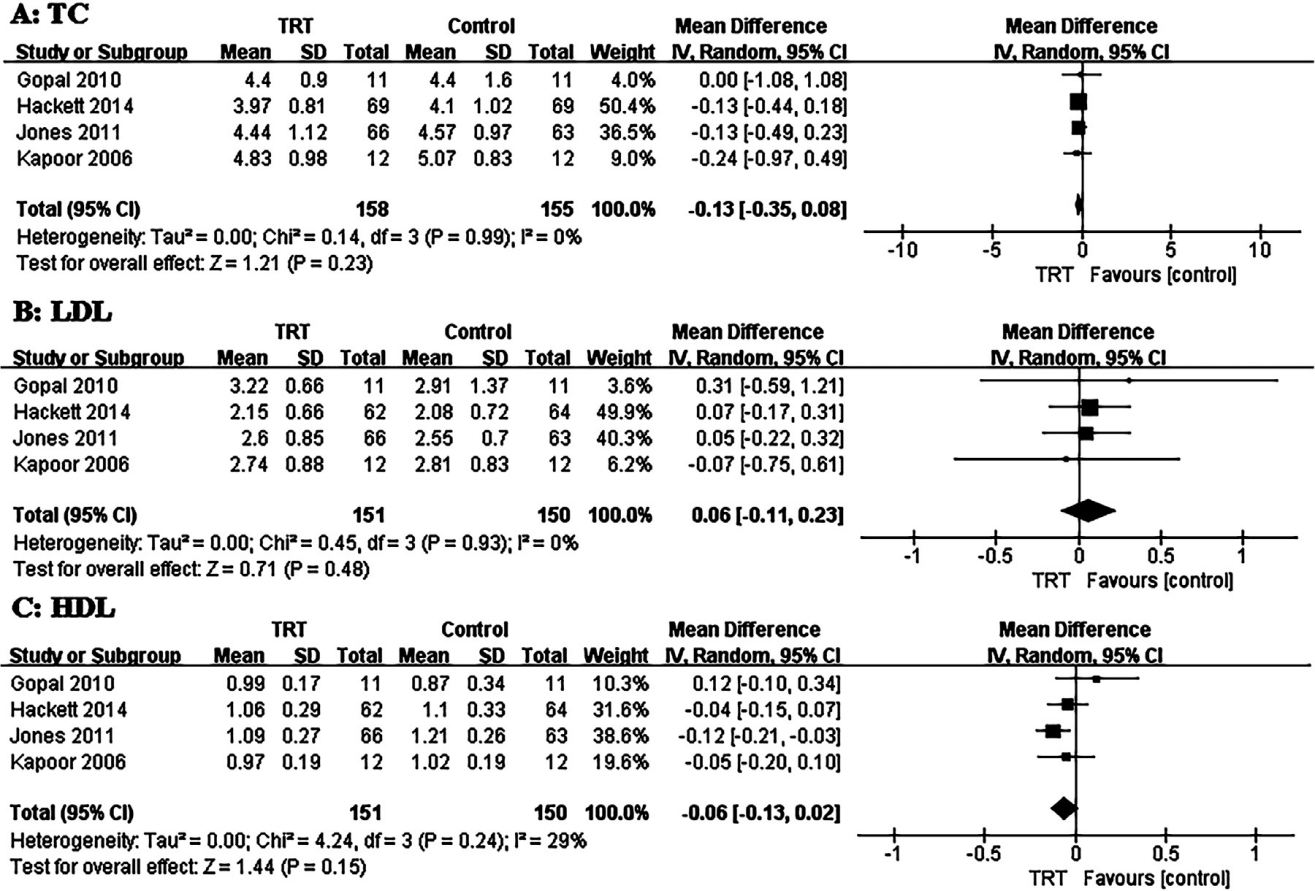
Weighted differences (with 95%CI) of mean Total Cholesterol (A:TC), low-density lipoprotein (B: LDL), and high-density lipoprotein (C: HDL) between testosterone and control groups. 385 × 270m (300 × 300 DPI).

For lipid metabolism, a pooled analysis was conducted with the outcomes of TC, HDL, and LDL. The TC pooled analysis included 313 patients, consisting of 158 in the TRT group and 155 as controls, resulting in a pooled WMD of−0.13,95% CI (−0.35, 0.08), *P* = .23 (Figure3A). For the LDL analysis, 345 patients were included, consisting of 170 who underwent TRT and 175 acting as controls, resulting in a pooled WMD of 0.06,95% CI (−0.11, 0.23), *P* = .48 (Figure3B). For the HDL analysis, 301 patients were included, consisting of 151 in the TRT group and 150 acting as controls, resulting in a pooled WMD of−0.06,95% CI (−0.13, 0.02), *P* = .15 (Figure3C).

**Figure 4:**
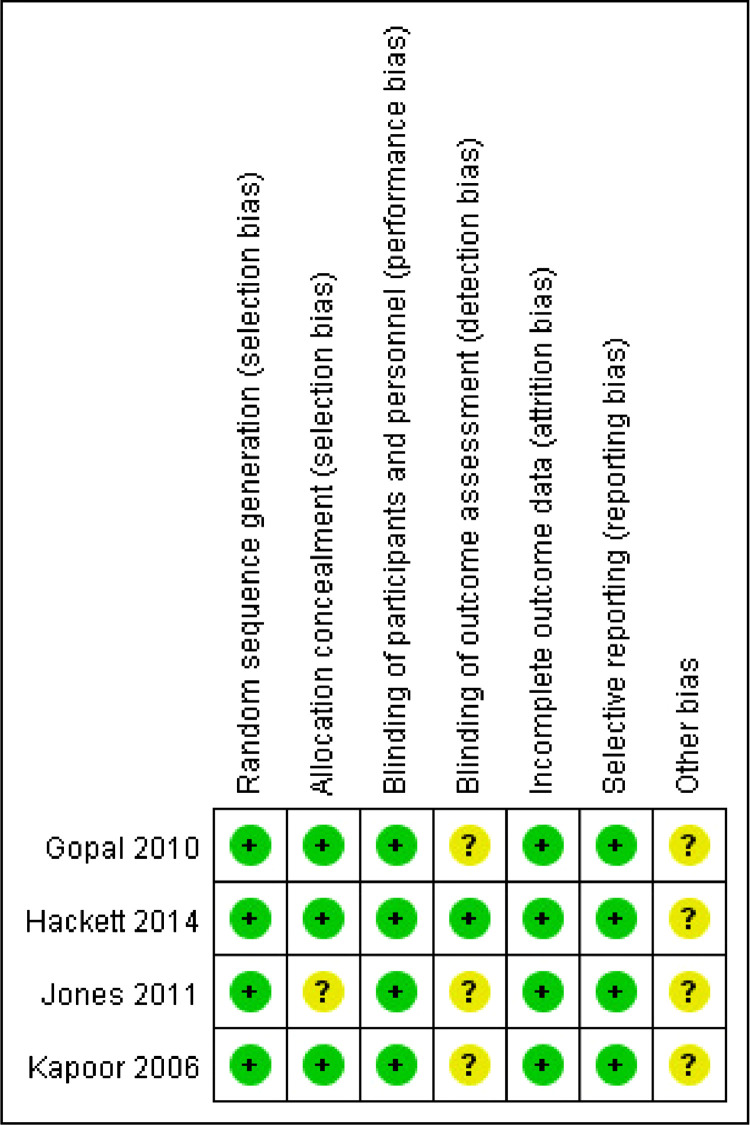
Risk of bias graph 7 × 11mm (600 × 600 DPI).

### Glycolipid Metabolism of TRT Monotherapy

In the remaining RCT by Khripun et al.,^19^ antidiabetic therapy in all participants was restricted to dietary changes only. Khripun et al.^19^ randomly assigned 80 hypogonadal men with T2DM to two groups, where they received either a 1%testosterone gel treatment or no treatment. Both groups also underwent a standardized diabetes diet for 36 weeks. Compared to the no treatment group, TRT was associated with significant improvements in FSG, FSI, HbA1c, HOMA-I, and HbA1c, as well as HDL. In Heufelder et al.’s study,^16^ 32 men with testosterone deficiency who were newly diagnosed T2DM were provided with a supervised diet and exercise, and they were also randomly assigned to either the TRT or no treatment group. The TRT group showed significant improvements in HOMA-I and in FSI and HbA1c as compared to the untreated group after one year, but not in any of the lipid metabolism parameters and FSG. Furthermore, 87.5% of patients who received TRT reached the HbA1c goal of less than 6.5%, whereas only 40.4% of the participants managed to reach a HbA1c of below 7.0%.

## DISCUSSION

Men with normal levels of fasting glucose have significantly higher levels of testosterone than those who are diagnosed with T2DM, and testosterone can improve glucose metabolism through multiple cellular mechanisms.^26^^,^^27^ Hypogonadal men were 2.3 times more likely to develop diabetes, thus hypogonadism might be an independent risk factor for diabetes.^28^ However, the administration of testosterone over a long period of 36 months in hypogonadal men did not seem to improve their insulin sensitivity.^29^ Hence, it remains unclear as to whether hypogonadal men with T2DM can benefit from TRT.

Many researchers have tried to investigate this issue from different perspectives. Currently published RCTs can be divided into three main categories. The most frequently used RCT design utilizes testosterone as the adjunctive therapy alongside insulin, conventional oral antidiabetics, antihypertensives, and lipid-lowering medications.^10^, ^11^, ^12^, ^13^^,^^15^^,^^18^ These papers report the synergistic metabolic effects of testosterone application and conventional treatment of diabetes. A second study design utilizes the adjunctive treatment of TRT alongside conventional oral antidiabetics, antihypertensives, and lipid-lowering medications, but insulin was not administered.^14^^,^^17^^,^^20^ The fact that insulin therapy was an exclusion criterion could be due to the need to clarify whether hypogonadal men with T2DM could avoid insulin use by improving their hypoandrogenic status. Since dual treatments combining TRT and antidiabetics were used, it is difficult to determine whether treatment outcomes were due to the effect of the TRT or the change in the diabetes treatment strategy during the course of treatment. Thus, it is important to ensure that conventional diabetes treatment strategies remain unchanged during the administration of the TRT as far as possible. However, in some studies, there were either changes in the type or dosage of the antidiabetic drug given during the course of the RCTs,^11^^,^^12^ or there was no mention of the consistency of the treatment.^10^^,^^14^ As these studies were incorporated into previous meta-analyses,^21^^,^^22^^,^^24^^,^^30^ the subsequent outcomes cannot be said to be solely the result of TRT, as it may also be due to the changes in treatment strategy.

Overall, the variability observed in treatment outcomes seems to be concentrated in studies without a consistent treatment strategy.^10^, ^11^, ^12^^,^^14^ Only one study^12^ concluded that TRT had no benefit on glycolipid metabolism among the four trials that had no consistent treatment strategy. The findings of the other three articles suggest that TRT reduces insulin resistance and improves glycemic control in hypogonadal men with T2DM,^10^^,^^11^^,^^14^ but the evidence available for this postulation is weak. For example, in Dhindsa et al.’s study,^11^ only a total of 14 patients and two participants in the control group changed their diabetic medication. In Groti et al.’s^14^ and Boyanov et al.’s^10^ studies, patients were only advised not to introduce any dietary or other life style changes during the study, but the articles make no mention of whether the dose and type of antidiabetic medication was changed throughout the study. Thus, the observed improvements may be due to patients receiving a more appropriate antiglycemic treatment strategy. In most of the trials that have maintained consistency in treatment strategies, TRT was found to be ineffective for improving glycemic control in hypogonadal men with T2DM,^13^^,^^15^^,^^20^ except in Kapoor et al.’s study,^18^ where 10 out of a total sample size of 24 participants were treated with insulin. Furthermore, Kapoor et al's study^18^ did not specifically describe which group these 10 patients were placed in, despite the fact that insulin is known to be better at maintaining blood glucose stability than other oral hypoglycemic agents.^31^

The third study design is used to clarify whether the use of TRT alone for hypogonadal men with T2DM without conventional diabetic medication can improve their metabolic profile. In this study design, TRT was used as a primary treatment rather than an adjunctive therapy, while the control group consisted of untreated men who either underwent a supervised diet or exercise only.^16^^,^^19^ These authors hypothesized that TRT might prevent or reverse early T2DM to a greater extent than a lifestyle program or diet alone. At the end of 36–52 weeks, although all patients showed improvements in HbA1c, fasting glucose, HDL levels, triglycerides, and waist circumference, the TRT group showed a greater improvement compared to the untreated group.^16^^,^^19^ Furthermore, 62.5% of patients in the TRT group no longer met the diagnostic criteria for T2DM, compared with 12.5% in the no treatment group.^16^ However, these studies should be treated as exploratory, as they included an untreated rather than a placebo control group.

The present systematic review and meta-analysis aimed to better assess the benefits of TRT on glucose metabolism in hypogonadal men with T2DM. Thus, we used a more rigorous inclusion criteria where additional treatment strategies such as antidiabetic medications, and lipid-lowering therapies had to remain consistent throughout the study period. Only four placebo-controlled, double-blind RCTs that met this inclusion criteria were included for this analysis.^13^^,^^15^^,^^17^^,^^18^ However, as the use of diabetes-related medications may be a confounder in the relationship between TRT and glucose metabolism outcome, there is insufficient evidence to suggest that TRT significantly improves glycolipid metabolism among hypogonadal men with T2DM.

In the included studies, participants mostly consisted of older men (mean age ranging from 56 to 64 years) who were obese (with a BMI range of 30–35 kg/m^2^). It is well known that testosterone levels in men are negatively correlated with BMI and age.^32^^,^^33^ Thus, unlike the other articles, Gopal et al.’s study^13^ that was included in our meta-analysis provided insights into the effect of TRT among T2DMmen who were not obese, as they included participants with BMIs within the normal range (Mean ± SD:23.94 ± 4.46). At the end of the 3-month experiment, TRT did not seem to result insignificant improvements in HOMA-I or glycolipid metabolism, and it also failed in controlling blood pressure or weight loss. There are several limitations to this systematic review. First, the assessment of testosterone levels and the cut-off values for defining hypogonadism varied between the RCTs included in the present meta-analysis. Second, the duration of treatment also varied among the different studies, ranging from 3 to 12 months. Although the design of these studies is consistent with the recommendation to reassess symptoms after 3 months of TRT,^2^ the difference in study duration may also lead to a discrepancy in the outcomes of the TRT. Third, the permissible use of various antidiabetics as well as the lack of information on the type and dose of antidiabetic medications administered by some of the included RCTs may have an impact on the reliability of the findings. Fourth, this review included RCTs with small sample sizes, which may not be enough to ascertain the actual effect of TRT.

## CONCLUSION

Although some RCTs showed a positive effect of TRTs on insulin resistance and glycolipid metabolism when compared with placebo or untreated controls, these findings may partly be due to changes in the antidiabetic medication used throughout the course of the study. The present meta-analysis thus demonstrated that TRTs did not significantly improve insulin resistance or glycolipid metabolism. However, the limitations related to this systematic review must be considered when evaluating the results of the present study. Future studies need to be rigorous in both their design and delivery, and include comprehensive descriptions of the methods used, as well as the patients’ situations, to enable a more accurate appraisal and interpretation of the results.

## Statement of Authorship

Conceptualization and Design: W.Q. and X.W.Y.; Acquisition of Data: X.Y., and Z.W.; Analysis: Q.W., and Y.L.; Drafting: X.Y., and Z.W.; Writing – Review & Editing, W.Q., Y.H.L., and X.Z.; Approval: X.Y., Z.W., Y.L., and Q.W..

## References

[1] Bhasin S, Cunningham GR, Hayes FJ (2010). Testosterone therapy in men with androgen deficiency syndromes: an Endocrine Society clinical practice guideline. J Clin Endocrinol Metabol.

[2] Buvat J, Maggi M, Guay A (2013). Testosterone deficiency in men: systematic review and standard operating procedures for diagnosis and treatment. J sex med.

[3] Kapoor D, Aldred H, Clark S (2007). Clinical and biochemical assessment of hypogonadism in men with type 2 diabetes: correlations with bioavailable testosterone and visceral adiposity. Diabetes Care.

[4] Grossmann M, Thomas MC, Panagiotopoulos S (2008). Low testosterone levels are common and associated with insulin resistance in men with diabetes. J clin endocrinol metabol.

[5] Stellato RK, Feldman HA, Hamdy O (2000). Testosterone, sex hormone-binding globulin, and the development of type 2 diabetes in middle-aged men: prospective results from the Massachusetts male aging study. Diabetes Care.

[6] Oh J-Y, Barrett-Connor E, Wedick NM (2002). Endogenous sex hormones and the development of type 2 diabetes in older men and women: the Rancho Bernardo study. Diabetes Care.

[7] Alibhai SM, Duong-Hua M, Sutradhar R (2009). Impact of androgen deprivation therapy on cardiovascular disease and diabetes. J Clin Oncol.

[8] Keating NL, O'Malley AJ, Smith MR (2006). Diabetes and cardiovascular disease during androgen deprivation therapy for prostate cancer. J Clin Oncol.

[9] Yassin A, Haider A, Haider KS (2019). Testosterone therapy in men with hypogonadism prevents progression from prediabetes to type 2 diabetes: eight-year data from a registry study. Diabetes Care.

[10] Boyanov M, Boneva Z, Christov V (2003). Testosterone supplementation in men with type 2 diabetes, visceral obesity and partial androgen deficiency. Aging Male.

[11] Dhindsa S, Ghanim H, Batra M (2016). Insulin resistance and inflammation in hypogonadotropic hypogonadism and their reduction after testosterone replacement in men with type 2 diabetes. Diabetes Care.

[12] Gianatti EJ, Dupuis P, Hoermann R (2014). Effect of testosterone treatment on glucose metabolism in men with type 2 diabetes: a randomized controlled trial. Diabetes Care.

[13] Gopal RA, Bothra N, Acharya SV (2010). Treatment of hypogonadism with testosterone in patients with type 2 diabetes mellitus. Endocr Pract.

[14] Groti K, Žuran I, Antonič B (2018). The impact of testosterone replacement therapy on glycemic control, vascular function, and components of the metabolic syndrome in obese hypogonadal men with type 2 diabetes. Aging Male.

[15] Hackett G, Cole N, Bhartia M (2014). Testosterone replacement therapy improves metabolic parameters in hypogonadal men with type 2 diabetes but not in men with coexisting depression: the BLAST study. J sex med.

[16] Heufelder AE, Saad F, Bunck MC (2009). Fifty-two-week treatment with diet and exercise plus transdermal testosterone reverses the metabolic syndrome and improves glycemic control in men with newly diagnosed type 2 diabetes and subnormal plasma testosterone. J Androl.

[17] Jones TH, Arver S, Behre HM (2011). Testosterone replacement in hypogonadal men with type 2 diabetes and/or metabolic syndrome (the TIMES2 study). Diabetes Care.

[18] Kapoor D, Goodwin E, Channer KS (2006). Testosterone replacement therapy improves insulin resistance, glycaemic control, visceral adiposity and hypercholesterolaemia in hypogonadal men with type 2 diabetes. Eur J Endocrinol.

[19] Khripun I, Vorobyev S, Belousov I (2019). Influence of testosterone substitution on glycemic control and endothelial markers in men with newly diagnosed functional hypogonadism and type 2 diabetes mellitus: a randomized controlled trial. Aging Male.

[20] Magnussen LV, Glintborg D, Hermann P (2016). Effect of testosterone on insulin sensitivity, oxidative metabolism and body composition in aging men with type 2 diabetes on metformin monotherapy. Diabetes, Obesity and Metabolism.

[21] Cai X, Tian Y, Wu T (2014). Metabolic effects of testosterone replacement therapy on hypogonadal men with type 2 diabetes mellitus: a systematic review and meta-analysis of randomized controlled trials. Asian J Androl.

[22] Corona G, Monami M, Rastrelli G (2011). Type 2 diabetes mellitus and testosterone: a meta-analysis study. Int J Androl.

[23] Grossmann M, Hoermann R, Wittert G (2015). Effects of testosterone treatment on glucose metabolism and symptoms in men with type 2 diabetes and the metabolic syndrome: a systematic review and meta-analysis of randomized controlled clinical trials. Clin Endocrinol (Oxf).

[24] Zhang J, Yang B, Xiao W (2018). Effects of testosterone supplement treatment in hypogonadal adult males with T2DM: a meta-analysis and systematic review. World J Urol.

[25] Chen B, Benedetti A (2017). Quantifying heterogeneity in individual participant data meta-analysis with binary outcomes. Systematic rev.

[26] Grossmann M (2011). Low testosterone in men with type 2 diabetes: significance and treatment. J clin endocrinol metabol.

[27] Gianatti E, Grossmann M (2020). Testosterone deficiency in men with type 2 diabetes: pathophysiology and treatment. Diabet Med.

[28] Laaksonen DE, Niskanen L, Punnonen K (2004). Testosterone and sex hormone–binding globulin predict the metabolic syndrome and diabetes in middle-aged men. Diabetes Care.

[29] Huang G, Pencina KM, Li Z (2018). Long-term testosterone administration on insulin sensitivity in older men with low or low-normal testosterone levels. J Clin Endocrinol Metab.

[30] Zhang KS, Zhao MJ, An Q (2018). Effects of testosterone supplementation therapy on lipid metabolism in hypogonadal men with T2DM: a meta-analysis of randomized controlled trials. Andrology.

[31] Holman RR, Thorne KI, Farmer AJ (2007). Addition of biphasic, prandial, or basal insulin to oral therapy in type 2 diabetes. N Engl J Med.

[32] Khera M, Adaikan G, Buvat J (2016). Diagnosis and treatment of testosterone deficiency: recommendations from the Fourth International Consultation for Sexual Medicine (ICSM 2015). The journal of sexual medicine.

[33] Haffner S, Valdez R, Stern M (1993). Obesity, body fat distribution and sex hormones in men. Int J Obes Relat Metab Disord.

